# ENDS retailers and marketing near university campuses with and without tobacco-free policies

**DOI:** 10.18332/tid/94600

**Published:** 2018-10-08

**Authors:** Dianne C. Barker, Nina C. Schleicher, Kimberly Ababseh, Trent O. Johnson, Lisa Henriksen

**Affiliations:** 1 Public Health Institute, Oakland, United States; 2 Stanford Prevention Research Center, Stanford School of Medicine, Palo Alto, United States

**Keywords:** ENDS retail marketing, electronic cigarettes, young adults, tobacco-free college campuses

## Abstract

**INTRODUCTION:**

This study characterizes the retail environment for Electronic Nicotine Delivery Systems (ENDS) near public universities in California, assesses marketing in the first random sample of ENDS retailers, and compares ENDS retailer density and retail marketing near campuses with and without tobacco-free policies.

**METHODS:**

Two data sources were used to construct a sampling frame of possible ENDS retailers, which were mapped within 1–4 miles of 33 campuses of the University of California and the California State University systems. To assess retailer density, a telephone survey of possible ENDS retailers (n=1186) determined who sold e-cigarettes or e-liquids (completion rate=72.9%). To assess retail marketing, trained data collectors completed observations in a random sample (n=438, mean M=13.3 stores per campus, SD=11.2) in the Fall of 2015

**RESULTS:**

In a telephone survey, 59.1% of retailers reported selling e-cigarettes or e-liquids. Half of the campuses had 10 or more ENDS retailers nearby. Most ENDS retailers were convenience stores (42.5%), and more were head shops (8.4%) than smoke shops (6.8%) or vape shops (6.2%). Nearly half (43.6%) of ENDS retailers sold products marketed as zero-nicotine and 13.9% sold NRT. ENDS advertising was visible in 72.4% and on the exterior of 28.1% of retailers. However, the presence of exterior advertising for ENDS was significantly lower near campuses with established tobacco-free policies than near campuses with recent or no tobacco-free policies (OR=0.45, 95% CI: 0.22–0.94).

**CONCLUSIONS:**

The large number of tobacco retailers that sell ENDS near colleges suggests a need for better monitoring and regulation of ENDS availability and marketing. The widespread availability of zero-nicotine products suggests a need to examine whether nicotine-free products are as advertised and safe to use. Longitudinal research is needed to understand how retail marketing for ENDS responds to change in tobacco-free policies at nearby campuses.

## INTRODUCTION

Experimentation with electronic nicotine delivery systems (ENDS) remains common, with approximately one-third of young adults reporting ever use in 2013–2014 in the National Adult Tobacco Survey (35.8%)^1^ and the U.S. Population Assessment of Tobacco and Health Survey (32.1%)^2^. Among U.S. college students, who tend to be early adopters of new products and a target market for the tobacco industry^3-4^, past-month ENDS use (8.8%) was almost as prevalent as cigarette smoking (11.3%) in the U.S. in 2015^5^. In the southeastern U.S., nearly half of a college-student sample (43.5%) reported trying e-cigarettes and 50% endorsed using e-cigarettes in places where smoking is banned as a reason for trying the product^4^. In Texas, college students who used ENDS exclusively were more likely to initiate cigarette smoking during college than students who never used ENDS^6^. Of concern is that ENDS use may increase the frequency, intensity and duration of subsequent cigarette smoking^7^.

ENDS also raise concerns about addiction potential in users who would otherwise be nicotine naïve^8^. While ENDS are less harmful than conventional (combustible) tobacco products, accumulating evidence about vaping highlights exposure to chemicals that are not considered safe for inhalation^9^, harmful emissions in exhaled vapor^10^, and product defects from overheating^11^.

The retail environment is for college students the primary source of exposure to marketing for all forms of tobacco^12^. Two studies observed higher odds of reporting past-month use of e-cigarettes among high-school students who attended schools in neighborhoods with a higher density of ENDS retailers specifically or tobacco retailers generally^13-14^. Higher density implies greater access to ENDS as well as exposure to pro-vaping cues. In a longitudinal study of college students who smoked cigarettes, greater exposure to ENDS retail displays, at baseline, was associated with lower odds of smoking abstinence at the 6 months follow-up^15^.

The only study, to date, of ENDS marketing near colleges was conducted near 11 campuses in North Carolina and Virginia^16^. The availability of disposable and rechargeable e-cigarettes more than doubled, the presence of exterior advertisements tripled, and the presence of interior advertising quadrupled within one year (2012–2013)^16^. By 2014, nearly all tobacco retailers sampled from a large Metropolitan Statistical Area in North Carolina sold ENDS (97.8%), with refillable tank systems and e-liquids sold in more than half of stores^17^. Building on this literature, this study is the first to characterize retail availability and marketing of ENDS in a random sample of ENDS retailers near public university campuses.

Tobacco-free campus policies may have direct effects on the retail availability and marketing of ENDS near college campuses. For example, 66.5% of U.S. college campuses had at least one vape shop within 3 miles in 2015, but the density of vape shops was significantly lower near campuses with a tobacco-free policy^18^. In independent samples of U.S. tobacco retailers, availability of ENDS was more common if retailers were located in counties or cities with the weakest smoke-free air laws (grade D or F) than in areas with the strongest smoke-free air laws (grade A)^19^. Retail patterns related to campus or local smoke-free policies may reflect lower demand for ENDS in communities where tobacco use is denormalized. The study compares retail environments near campuses with and without tobacco-free policies by taking advantage of an on-going natural experiment in the public university system in California.

The current study advances research about the retail environment for ENDS by: 1) conducting a telephone survey to assess the quantity of tobacco retailers that sold ENDS near all four-year public universities in California; 2) conducting retail marketing surveillance to assess product availability, placement and promotion in a random sample of ENDS retailers; and 3) examining differences in the retail density and marketing for ENDS as a function of campus policies that restrict tobacco use.

## METHODS

We first constructed a sampling frame of tobacco retailers near the 33 public university campuses in California: 13 of the University of California (UC) and 20 of the California State University (CSU). Data collection procedures for a telephone survey of retailers were followed by in-store observations of marketing. All data were collected in the Fall of 2015. Institutional Review Boards of Stanford University School of Medicine and of the Public Health Institute determined that this study protocol did not constitute human subjects research.

### Sampling frame

California has required a State retail license for the sale of conventional tobacco products since 2004. The State did not require a license for stores that sold ENDS exclusively until 2017. In an effort to enumerate retailers that were not yet licensed, we adapted the Kim et al.^20^ protocol. We used their list of keywords (i.e. ecig, e-cigarette, vape, vapor, vaper, vapin’) and three additional terms (e-juice, e-liquid, electronic cigarette) to scrape data from Yelp, Google and Yellow Pages websites. After removing duplicate entries and matching the scraped list of the 1922 potential vape shops in California with a 2015 State licensing list of 34428 tobacco retailers, we found that 632 retailers were already licensed. Thus, the scraped list yielded an additional 1290 retailers that were not yet licensed, for a combined list of 35718 possible ENDS retailers in the State.

Using ArcGIS v10.1, we geocoded all of the retailer addresses (mapping rate=99.4%) and joined these points with campus boundary shapefiles that we obtained or created^21^. We created campus neighborhoods by drawing a 1-mile (Euclidean) buffer from each campus boundary for 30 urban/suburban campuses^22^ and a 4-mile buffer from boundaries of three rural campuses. A total of 1186 potential ENDS retailers were located within these 33 campus neighborhoods.

### Telephone survey to assess ENDS retailer density

Our research team trained professional data collectors to telephone all possible ENDS retailers (n=1186) in the campus neighborhoods. Yelp and Google were used to update telephone numbers from the State tobacco retail licensing list and the scraped list of vape shops. The telephone script confirmed the store name, asked if the store sold ‘e-cigs or e-juice’, and prompted with specific brand examples as needed (e.g. ‘Do you sell electronic cigarettes like Blu, NJOY, or Vuse?’). After removing 172 ineligible records (e.g. wrong number, disconnected, residence or permanently closed), the completion rate for the telephone survey among eligible stores was 72.9% (739/1014). Reasons for non-completion were no answer (10.9%), an answering machine that did not return a message (10.7%), fax line (3.6%) and other (1.6%) (e.g. refusal, language barrier).

### Store observations to assess ENDS retail marketing

#### Sample

Data collectors attempted to visit: all stores that reported selling ENDS (n=437), three vape shops from the telephone verification sample that could not be reached by telephone but were active on social media, and 60 licensed retailers with incomplete telephone verifications. Of the 62 stores that were incomplete, 52 were ineligible, i.e. the stores did not sell ENDS (n=43) or did not exist (n=9), 8 were temporarily closed and 2 refused. The completion rate among eligible stores was 97.7% (438/448). Data collectors completed observations in an average of 13.3 stores per campus (SD=11.2, min=2, max=63).

#### Data collection protocol

Four professional data collectors participated in a full day of in-person instruction to assess ENDS retail marketing (i.e. product availability, placement and promotion). Instruction included field practice in multiple stores and interactive quizzes to assess adherence to protocol. Data collectors also participated in a teleconference, after the first days of surveillance in the field, to review questions and clarify instructions. Each store was visited by one of four data collectors and retail observations averaged 12 minutes per store. The 65-item instrument that was included assessed the following constructs.

#### Store type

Data collectors initially classified stores into one of eight categories using standard definitions for conventional tobacco retailers: convenience store, liquor, pharmacy, supermarket, small market, smoke shop, discount store and other^23^. A category for vape shops was defined as a retailer primarily engaged in the sale of ENDS, with at least 50% of merchandise related to ENDS^24^. The few discount stores (n=4) and supermarkets (n=10) were merged into ‘other’, resulting in eight categories for analysis that included: convenience, liquor, pharmacy, small market, smoke shop, vape shop, and head shop (a separate category, although it was not defined a priori). Data collectors described more than half of stores in the ‘other’ category as head shops.

#### Product availability

Data collectors assessed the presence of e-cigarettes (closed systems that were pre-filled) and other vaping devices (open systems that users could fill with e-liquid), including vape pens, mods, e-hookah and e-cigars). Presence of e-cartridges and e-liquids was also recorded. In addition, data collectors reported whether stores sold e-liquids or e-cigarettes marketed as nicotine-free or ‘zero’ nicotine. They recorded availability of cessation-related products: nicotine replacement therapy (e.g. NRT gum, patch, or lozenges approved by the U.S. Food and Drug Administration), and separately recorded the presence of Zonnic, a nicotine gum marketed by Niconovum/RJ Reynolds. Whether stores sold each of four conventional tobacco products, i.e. cigarettes, little cigars/cigarillos, large cigars and hookah (pipes and/or shisha tobacco), was also noted.

#### Product placement and promotion

Data collectors indicated whether ENDS products were available in self-service displays or placed on a front counter. In addition, data collectors recorded interior and exterior advertising by noting the presence of any branded signs, shelving units, displays, and functional items. These data were collected for ENDS as well as for cigarettes and little cigars/cigarillos (LCCs).

### Campus tobacco-free policies and enrollment

We linked data about ENDS retail density and marketing near college campuses with data about tobacco-free policies at the 33 university campuses. All 10 UC campuses had implemented a tobacco-free policy that prohibits smoking and vaping on campus since 1 January 2014. Although the CSU system was charged with adopting a system-wide policy by the same deadline, no statewide policy had been declared. The few tobacco-free policies at CSU campuses similarly restricted smoking and vaping. Combining policy ratings prepared by the California Youth Advocacy Network with the policy implementation date^25^, we categorized the 33 campuses as: 1) having an established policy because they were tobacco or smoke-free for at least one year prior to data collection, 2) having a recent policy because they were tobacco-free or smoke-free for less than one year prior to data collection, or 3) not being smoke-free because they had limited restrictions on smoking and other tobacco use or had no policy at all. All universities that had tobacco-free policies included e-cigarettes. There were no substantial differences between tobacco-free policies other than time since implementation. Tobacco-free campus policies typically forbid the sale of tobacco products on campus and there were no tobacco retailers on campuses.

For each campus, we obtained student enrollment from the Integrated Postsecondary Education Data System^26^. Measures included number of students enrolled full time, per cent of students receiving financial aid, and race/ethnicity (Hispanic, non-Hispanic African American, non-Hispanic Asian/Pacific Islander, non-Hispanic White, and multiple races/unknown). All measures pertained to the undergraduate population, unless campuses served graduate students only (e.g. University of California, San Francisco).

### Analyses

#### ENDS retailer density

For each campus neighborhood we computed the number of telephone-verified ENDS retailers divided by the number of enrolled full-time undergraduate students (graduate students for UCSF). The distribution of retailer density (ENDS retailers per 1000 students) was positively skewed (skewness=5.4); therefore, we compared the median and IQR by campus policy rather than the mean and standard deviation. For a sensitivity analysis, we computed the number of verified ENDS retailers divided by the land area (square miles) within the campus neighborhood.

#### ENDS retail marketing

To characterize product availability, we generated descriptive statistics overall and by store type for five categories of ENDS: e-cigarettes, any other vaping device (including vape pens, mods, e-hookah and e-cigars), e-cartridges, e-liquids, and specifically zero-nicotine e-liquids or e-cartridges. For comparison, we also summarized the availability and marketing of conventional tobacco products and nicotine replacement therapy. In addition, we generated percentages overall and by store type for product placement (i.e. on front counter and self-service), and for any ENDS and cigarette/LCCs advertising by location (exterior and interior). Chi-squared tests were used to examine differences by store type.

Generalized linear mixed models with retailers (level 1, n=438) nested in campus neighborhoods (level 2, n=33) were fit for two dichotomous outcomes: any exterior advertising for ENDS and for cigarettes/LCCs. The models examined the two outcomes as a function of tobacco-free policy and campus enrollment characteristics and controlled for store type. Enrollment characteristics (number of students, % receiving financial aid, and race/ethnicity) were standardized in models to yield meaningful coefficients. Race/ethnicity was modeled as the per cent of non-Hispanic White because of concerns about overparameterization due to the small number of level two units (n=33). Population average models with a random level 1 intercept and robust standard errors were fit using HLM7.0 software.

## RESULTS

Table 1 summarizes the characteristics of student enrollment, tobacco-free campus policies, and ENDS retailer density in the campus neighborhoods (e.g. within 1 mile of the campus boundaries and 4 miles of the few rural campuses). Only 12 of the 33 campuses had instituted tobacco-free policies at least one year prior to 2015 and three campuses implemented tobacco-free policies within 12 months prior to the 2015 data collection. Campuses with tobacco-free policies had higher student enrollment and a higher percentage of students who were Asian/Pacific Islanders than campuses without tobacco-free policies (Table 1).

**Table 1:** Characteristics of California public university campuses (n=33) and density of ENDS retailers (n=438) per 1000 students

	*Tobacco-free ≥1 yr n=12*		*Tobacco-free <1 yr n=3*		*Not tobacco-free n=18*		*Overall n=33*	

	*Mean*	*SD*	*Mean*	*SD*	*Mean*	*SD*	*Mean*	*SD*
Student body*								
Enrolled, full time	22438	9737	23558	13403	15594	8843	18807	9911
**Race/Ethnicity**, %								
Hispanic	24.8	10.3	29	9.2	35.6	13.2	31.1	12.7
Non-Hispanic African American	3.1	1.6	4	2.1	5	3.7	4.2	3
Non-Hispanic Asian/Pacific Islander	29.4	9.6	17.2	16.4	13.4	8.1	19.5	11.9
Non-Hispanic White	27.4	8.5	33.7	17.2	30.4	15.6	29.6	13.3
Multiple races/unknown race	15.3	4.5	16.1	1.3	15.5	3.3	15.5	3.6
**Receive financial aid, %**	66.1	12.1	49.4	8.3	59	9.5	60.7	11.3
	Median	IQR	Median	IQR	Median	IQR	Median	IQR
**Density of ENDS retailers**	0.5	0.6	0.7	N/A	0.7	1.5	0.7	0.7

Density = retailers per 1000 students within 1-mile buffer of urban/suburban campuses and 4-mile buffer of rural campuses. IQR: interquartile range.

* Undergraduate population, except UCSF.

### ENDS retailer density and tobacco-free campus policies

Of the 739 stores with complete telephone verifications, 59.1% reported selling e-cigarettes or e-liquids. The median number of ENDS retailers per campus neighborhood was 10 (IQR=11.5). Overall, the median density of ENDS retailers near campuses was 0.7 per 1000 students (IQR=0.7). Contrary to expectation, ENDS retailer density did not differ by campus tobacco-free policy although density was in the expected direction: the median density was 0.5 (IQR=0.6) for campuses that were tobacco-free for at least one year (n=12), 0.7 (IQR=N/A) for campuses that were tobacco-free for less than one year (n=3), and 0.7 (IQR=1.5) for campuses that had no tobacco-free policy (n=18). Density did not differ by campus policy for the sensitivity analysis (data not shown).

### ENDS retail marketing and tobacco-free campus policies

Using data from the marketing surveillance research, Table 2 illustrates the composition of ENDS retailers near the 33 public university campuses in California. Convenience stores were the most prevalent ENDS retailers (42.5%) followed by liquor stores (13.7%), small markets (10.3%), head shops (8.4%), pharmacies (7.1%) and smoke shops (6.8%). Vape shops represented only 6.2% of ENDS retailers near college campuses.

**Table 2:** ENDS and tobacco product availability near university campuses, by store type: California, 2015 (n=438)

			*ENDS Product Availability, %*				*Other Tobacco Product Availability, %*				*NRT, %*

	*n*	*E-cigs*	Any other vaping device*	*E-cartridges*	*E-liquid*	Zero-nicotine e-liquid or e-cigs**	*Cigarettes*	*LCCs*	*Large cigars*	*Hookah (pipe and tobacco)*	*FDA-approved cessation products*
**Store type**											
Convenience	186	98.4	36.6	81.7	36.6	30.1	100.0	98.4	30.1	17.7	11.3
Liquor	60	95.0	31.7	68.3	38.3	31.7	100.0	95.0	65.0	35.0	0.0
Pharmacy	31	100.0	80.6	100.0	80.6	32.3	100.0	100.0	96.8	22.6	100.0
Small market	45	97.8	26.7	64.4	24.4	22.2	95.6	95.6	20.0	8.9	0.0
Smoke shop	30	100.0	93.3	96.7	96.7	93.3	100.0	100.0	96.7	96.7	3.3
Vape shop	27	40.7	92.6	44.4	85.2	100.0	37.0	3.7	3.7	3.7	0.0
Head shop	37	83.8	100.0	75.7	100.0	94.6	86.4	83.8	54.1	89.2	0.0
Other	22	95.5	31.8	36.4	31.8	27.3	73.0	72.7	36.4	31.8	36.4
**Total**	438	93.2	50.5	75.3	50.9	43.6	90.6	89.5	43.8	30.8	13.9
p-value for chi-squared test		<0.001	<0.001	<0.001	<0.001	<0.001	<0.001	<0.001	<0.001	<0.001	<0.001

NRT: Nicotine Replacement Therapy. The few discount stores (n=4) and supermarkets (n=10) were merged into ‘other’, resulting in eight final store types.

* Any other vaping device includes vape pens, mods, e-hookah and e-cigars.

** Zero-nicotine e-cigarettes refers to closed system e-cigs. LCCs: little cigars/cigarillos.

### Product availability

Table 2 indicates that closed system e-cigarettes were the most widely available ENDS product followed by e-cartridges. Half of the ENDS retailers sold e-liquids and open tank systems. These were more commonly found in specialty shops, such as head shops (100%), smoke shops (93.3-96.7%) and vape shops (85.2-92.6%) as well as pharmacies (80.6%). ENDS marketed as zero-nicotine products (e-liquids and in some stores, e-cigarettes) were sold in 43.6% of stores, available in almost all specialty shops, but in less than one-third of other retailers. FDA-approved nicotine replacement therapies (NRT) were available: in only 13.9% of ENDS retailers, including all pharmacies, in 36.4% of other stores, in 11.3% of convenience stores, and in one smoke shop.

As shown in Table 2, most ENDS retailers near campuses sold cigarettes (90.6%) and LCCs (89.5%) and nearly half sold large cigars (43.8%). Hookah (pipes or shisha tobacco) was sold in 30.8% of ENDS retailers near campuses. Among the 27 vape shops, only one sold any conventional tobacco products.

### Placement and promotion

Self-service displays for ENDS were found in 3.0% of retailers. ENDS were displayed on the front counter in approximately 4 in 10 retailers (Table 3). These front-counter displays were common in liquor stores (63.3%), vape shops (74.1%), head shops (70.3%) and smoke shops (63.3%).

**Table 3:** Product placement and presence of advertising of ENDS and other tobacco products, by store type (n=438)

	*ENDS Product Placement*			*ENDS Advertising*		*Cigarette/LCC Advertising*		

	*n*	*Any ENDS self-service%*	*Any ENDS on the front counter%*	*Any interior ENDS advertising%*	*Any exterior ENDS advertising%*	*n*	*Any interior cigarette/LCC advertising%*	*Any exterior cigarette/LCC advertising%*
**Store type**								
Convenience	186	2.2	29.0	66.8	26.9	186	94.1	51.1
Liquor	60	3.3	63.3	51.4	25.0	60	86.7	38.3
Pharmacy	31	3.2	0.0	17.6	0.0	31	77.4	0.0
Small market	45	2.2	46.7	75.1	17.8	44	86.4	27.3
Smoke shop	30	13.3	63.3	44.4	63.3	30	93.3	76.7
Vape shop	27	3.7	74.1	13.7	7.4	1	0.0	0.0
Head shop	37	0.0	70.3	79.5	75.7	31	93.5	54.8
Other	22	0.0	22.7	10.1	4.5	19	47.4	10.5
**Total**	438	3.0	41.8	72.4	28.1	402	88.3	42.8
p-value for chi-squared test		<0.001	<0.001	<0.001	<0.001		<0.001	<0.001

Statistics about cigarette/LCC advertising are for a subgroup of stores that sold cigarettes or LCCs (n=402) instead of full sample (n=438).

Exterior and interior advertising for ENDS was somewhat less prevalent than advertising for any cigarettes or LCCs, as shown in Table 3. With the exception of vape shops, pharmacies and other stores, most retailers (72.4%) displayed interior advertisements for ENDS products and almost all retailers (88.3%), except vape shops, had interior advertisements for cigarettes/LCCs. Far fewer retailers advertised ENDS (28.1%) or cigarettes/LCCS (42.8%) on the store exterior.

Table 4 summarizes results from adjusted generalized linear mixed models for ENDS and other tobacco product marketing on the store exterior. Stores near campuses with a more established tobacco-free policy (≥1 year) were significantly less likely to advertise ENDS (OR=0.45, p=0.03) than stores near campuses with a recent policy or no tobacco-free policy at all. As shown in Table 4, smoke shops and head shops were significantly more likely to have exterior ENDS marketing than all other store types combined (e.g. convenience, liquor, small markets and pharmacies). The presence of ENDS advertising at vape shops did not differ from the combination of other store types.

**Table 4:** Correlates of exterior advertising for ENDS and tobacco products from generalized linear mixed models

	*Model 1 Any exterior ads for ENDS*			*Model 2 Any exterior ads cigarettes/LCCs*		

	*OR*	*p*	*95% CI*	*OR*	*p*	*95% CI*
Intercept	0.34	<0.001	(0.22–0.54)	0.77	0.16	(0.53–1.12)
**Stores (Level 1)**	n=438			n=402		
Store types						
All other store types	reference			reference		
Smoke shop	7.14	<0.001	(3.07–16.59)	4.74	<0.001	(2.23–10.07)
Vape shop	0.30	0.25	(0.04–2.34)	N/A		
Head shop	11.97	<0.001	(4.59–31.22)	2.08	0.07	(0.95–4.58)
**Campus (Level 2, n=33)**	n=33			n=33		
Enrollment size	1.07	0.77	(0.67–1.71)	1.06	0.73	(0.76–1.47)
% Financial aid	1.38	0.24	(0.80–2.38)	1.03	0.88	(0.69–1.53)
% White	1.17	0.61	(0.64–2.14)	1.21	0.30	(0.84–1.75)
Campus Policy						
Not tobacco-free	reference			reference		
Tobacco free <1 year	0.97	0.91	(0.50–1.87)	0.82	0.62	(0.37–1.82)
Tobacco free at least 1 year	0.45	0.03	(0.22–0.94)	0.74	0.33	(0.40–1.37)

Odds ratios are adjusted for all other variables in the model. Vape shops are not included in Model 2 due to small sample size (n=1 for Cigarette/LCC model). Cigarettes/LCC model refers to subset of stores that sold cigarettes and/or LCCs (n=402). Enrollment characteristics (number of students, % receiving financial aid, and race/ethnicity) were standardized.

## DISCUSSION

This study is novel in constructing a random sample of ENDS retailers. According to the telephone survey, approximately 6 in 10 tobacco retailers near public university campuses in California reported selling e-cigarettes or e-liquids. The majority of ENDS retailers were convenience stores, and more head shops than smoke shops or vape shops sold ENDS near the campuses. We hypothesized a lower density of ENDS retailers near tobacco-free campuses because prior research found a lower density of vape shops near smoke-free college campuses^18^. The estimated result for ENDS retailer density was in the hypothesized direction, but was not statistically significant.

This study contributes to our understanding of ENDS marketing near college campuses. Although refillable vaping devices are more popular among young adults and adolescents than disposable e-cigarettes^27^, the latter were still the most widely available ENDS product. Four in 10 stores displayed ENDS on the front counter, presumably to increase visibility and encourage impulsive buying^28-29^. Although students and staff at campuses with smoke-free policies may have greater interest in smoking cessation, the retail availability of NRT was surprisingly low, sold in only 13.9% of ENDS retailers. Nearly half of ENDS retailers sold e-cigarettes, cartridges or e-liquids labeled as zero-nicotine or nicotine-free, which is concerning because some products labeled nicotine-free contain some nicotine^30^. Although rare, ENDS displayed as self-service products (in 3% of stores) constitute a violation of California State law^31^.

College students may be susceptible to ENDS marketing, given the evidence that suggests their uptake of ENDS is more for enjoyment than for quitting smoking^32^. Near the 33 public university campuses in California, the prevalence of ENDS marketing was comparably higher than in tobacco retailers near colleges^16^ in North Carolina and Virginia in 2013. In this California sample, the presence of interior advertising for ENDS was almost as ubiquitous as interior advertising for cigarettes/LCCs. Advertising for cigarettes/LCCs was so prevalent that we did not detect any associations of exterior marketing for these products with either campus policies or characteristics of the student enrollment. Regardless of campus policy, many campuses are surrounded by a retail environment that promotes ENDS and conventional tobacco products. The only significant difference in stores near campuses with established tobacco-free policies compared to other stores was a lower likelihood of having exterior advertising for ENDS. This new finding could reflect lower demand for ENDS in campuses or communities with stronger anti-tobacco norms. However, the underlying cause requires further investigation. Future research could investigate whether differential marketing patterns are related to campus policies or attributable to other factors, such as the demography of students or nearby residents.

Strengths of this study are the combination of data for licensed tobacco retailers and a list of vape shops from online data (unlicensed retailers) to create a sampling frame of potential ENDS retailers. This study is first to characterize all types of retailers that sell ENDS near college campuses and how the products are marketing in a large sample of stores near all four-year public universities in a large and racially diverse State. Limitations are the response rate for the telephone survey, which may have underestimated the retail availability of ENDS. In addition, the data collection vendor inadvertently forgot to schedule repeat visits by independent data collectors, which made it impossible to assess inter-rater reliability. However, previous studies with similar protocols have obtained reliable assessments of product availability, product placement, and promotion^19,33^. The small number of campuses made it more difficult to detect associations between ENDS retailer density/marketing and campus tobacco-free policies. In addition, we did not have information about the enforcement of tobacco-free policies on campuses to include as a potential confounder.

Retail marketing surveillance is particularly important given the rapid uptake of tobacco-free colleges in the U.S.^34-35^ as well as local regulation of vape-free air and State deregulation of marijuana. Retail policies are much needed to prevent nicotine use and dependence among the next generation of college students^36^. Beyond increasing the minimum legal sales age to 21 years, States and local jurisdictions could include campuses for postsecondary education in efforts to limit the sales of any/flavored tobacco retailers near schools. Such policies would reduce retail access and exposure to pro-tobacco cues among young adults. Reducing the visibility of ENDS products could be hastened by extending federal self-service bans to include ENDS products and promoting voluntary merchant programs to store all tobacco products out of view. Given the widespread availability of products marketed as nicotine-free, States need to identify a regulatory mechanism to confirm such products are as advertised and safe for use. With regard to retail environments near college campuses, youth advocacy programs like the California Youth Advocacy Network and Texas College Tobacco Project *Peers Against Tobacco* can serve as models for collaboration, among students, campus administrators, and retailers near a campus, on the need for retail policies to complement tobacco-free campus policies and the importance of ongoing enforcement.

## CONCLUSIONS

Surveillance of the retail environment for ENDS is essential to understand how tobacco companies and other manufacturers distribute and market ENDS as well as how industry responds to tobacco control policy changes on campus, in communities, and in States. More than half of tobacco retailers near California public universities reported selling ENDS in 2015. The median number of ENDS retailers in the campus neighborhoods was 10 (IQR=11.5), with a median density of 0.7 ENDS retailers per 1000 students at the nearby university campus. The large number of tobacco retailers that sell ENDS near colleges suggests a need for better monitoring and regulation of ENDS availability and marketing.

The fact that exterior advertising for ENDS was significantly lower near campuses with established tobacco-free policies than near campuses with recent or no tobacco-free policies is a new finding. Longitudinal studies with larger samples of college campuses and the nearby ENDS retailers are needed to better understand how retail marketing contributes to product initiation and use patterns among college students and whether campus tobacco-free policies moderate this impact.
